# Comparison and concordance of health-related quality of life tests among substance users

**DOI:** 10.1186/s12955-015-0364-8

**Published:** 2015-11-19

**Authors:** Antonio J. Rojas, Oscar Lozano, Katia Foresti, Elham Zolfaghari, Carlos Zubaran

**Affiliations:** Department of Psychology, University of Almería. Facultad de Psicología, 04120 Almería, Spain; Department of Clinical, Experimental and Social Psychology, University of Huelva, Facultad de Ciencias de la Educación, 21071 Huelva, Spain; Department of Psychiatry, Hornsby Hospital, Northern Sydney Local Health District, Palmerston Rd, Hornsby, NSW 2077 Australia; Department of Psychiatry, Blacktown Hospital, Western Sydney Local Health District, PO Box 762, Seven Hills, NSW 2147 Australia; School of Medicine, Western Sydney University, PO Box 6010, Blacktown, NSW 2148 Australia

**Keywords:** Health-related quality of life, Health-related quality of life test, Substance users, Score linkage, Concordance, Rasch models

## Abstract

**Background:**

In the field of drug and alcohol abuse, health-related quality of life (HRQoL) has been used as an important clinical and research outcome. The aim of this study was to establish score linkages (concordance) among three HRQoL assessment tools: WHOQOL-BREF, DUQOL and HRQOLDA scores, applying a Rasch-based common person equating procedure.

**Methods:**

One hundred and twenty one adults were recruited from inpatient and outpatient treatment facilities in Sydney West Area Health Service. WHOQOL-BREF, DUQOL and HRQOLDA tests were administered. Item parameters were calculated applying Rating Scale Model, a Rasch model.

**Results:**

Fit statistics suggest acceptable goodness-of-fit to the RSM for three instruments. Correlations between HRQOLDA and WHOQOL-BREF and between HRQOLDA and DUQOL scores were 0.719 and 0.613, and the RiU index was 30.4 % and 20.9 %, respectively. All three tests performed adequately for differentiating between individuals whose scores are located at different points along the continuum of the HRQoL construct.

**Conclusion:**

The results demonstrated a higher concordance between the HRQoLDA and WHOQOL-BREF than between the HRQoLDA and the DUQOL. However, it cannot be established unequivocally that the scores of these tools are concordant. In this study, the utility of the application of the Rasch model to provide an empirical benchmark for the selection of measurement tools to be used in the context of health care and research is demonstrated.

## Background

In the field of drug and alcohol abuse, HRQoL has been used as an important clinical and research outcome. HRQoL started to be used as a complementary outcome measure to “hard” indicators, such as criminal behaviours, unemployment, and illicit drug use [1]. The concept of HRQoL is also used in drug abuse research and clinical practice, including the assessment of the impact of therapeutic interventions on patient’s lives [2, 3].

In the field of substance use, HRQoL has been measured mainly via generic tests. These tests evaluate representative behaviours from daily activities and most frequent symptoms in normal persons. The World Health Organization Quality of Life (WHOQOL-BREF) is a generic questionnaire, which has been used widely as assessment tool in epidemiological research. The WHOQOL-BREF has been translated to various languages and tested internationally [4, 5].

In recent years, specific tests have been developed to assess quality of life in the context of substance use, including the Injection Drug Users Quality of Life Scale (IDUQOL) [6, 7]; the Quality of Life Scale for Drug Addicts (QOL-DA) [8]; and the Health-Related Quality of Life for Drug Abusers test (HRQOLDA) [9, 10]. By contrast, the Drug User Quality of Life Scale is a specific instrument, which was originally developed to assess the quality of life of intravenous substance users [6, 7]; a later version has been adapted for substance users irrespective of their methods of self-administration (DUQOL) [11]. This instrument was conceptualized using the World Health Organization’s definition of quality of life. There are versions of the DUQOL available both in English [6, 7] and Spanish [11], and both present sound psychometric properties of reliability and validity – statistical analyses revealed an ICC score of 0.71 [6], a Cronbach's alpha coefficient of 0.86 and a test-retest reliability of 0.79 [11], with favorable evidence of convergent validity [6], content validity evidence [7], and criterion-related validity [11]).

The HRQoLDA [10] or TECVASP test as originally conceived in the Spanish language [9], as a quality of life instrument designed to specifically assess the quality of life of substance users. This specific instrument was developed to assist clinicians and researchers to determine to what extent and intensity substance use and drug addiction affect Quality of Life (QoL). The HRQoLDA is a brief assessment tool, which measures aspects related to the physical, psychological and social consequences of substance use. The HRQoLDA is a reliable instrument for evaluating quality of life of substance users, which has demonstrated a significant Cronbach's alpha coefficient of 0.905, with sound evidence of convergent validity [10].

Numerous methods have been developed over the years for correlating measurement tests and scales. The term *linking* refers to the general class of transformations between the scores from one test to another [12]. Three main methodologies have been proposed to select the best applicable score linkage to a given scenario, which are *equating*, *scale aligning*, and *prediction* [13]. The use of any of these approaches depends on construct similarity between tests, as well as difficulty, reliability, and constancy of the linkage relationship across populations [14]. Equating is the strongest form of linking between test scores, whereas prediction is considered the weakest. Concordance, a scale aligning subcategory, is used in tests that measure similar constructs according to different blueprints or test content specifications [15]. Concordance is a form of linking to establish score comparability from different tests that measure ideally similar, but not necessarily equivalent constructs, to be used approximately in the same way and given similar interpretations [16]. Concordance represents scaling of tests that were not created with the idea that their scores would be used interchangeably [15].

The WHOQOL-BREF, DUQOL and HRQoLDA are different tests that measure HRQoL construct, but their operational definitions are distinct. However, the three tests are currently used to assess HRQoL in the field of addiction. In this study, the authors present the results of analyses to assess the concordance of WHOQOL-BREF, DUQOL and HRQoLDA scores via a Rasch-based common person equating procedure. Common person equating procedure is conventionally used to evaluate different tests administered to a common group of research participants. The specific aim of this study was to establish score linkages (concordance), using a Rasch-based common person equating procedure, between the DUQOL and HRQoLDA and between WHOQOL-BREF and HRQoLDA.

## Methods

### Sample

This study evaluated 121 adults recruited from inpatient and outpatient treatment facilities across the Sydney West Area Health Service (SWAHS) catchment area in western Sydney, Australia. Research sites included Blacktown Hospital, Cumberland Hospital, Nepean Hospital, and the Mount Druitt Centre for Addiction Medicine, all of which are higher education training facilities within Sydney West Area Health Service (SWAHS).

Potential participants presenting for treatment were randomly invited to respond to the questionnaires. The inclusion criteria consisted of fulfilment of the DSM-IV criteria for substance abuse disorders (substance dependence and abuse disorders) and the ability to understand the aim of the study, as well as the content of the questions in both questionnaires, which entailed a satisfactory command of English. Exclusion criteria comprised of presentations exclusively due to alcohol abuse and/or involuntary admission for inpatient treatment.

This study was granted approval by the SWAHS Human Research Ethics Committee. Prospective participants were provided with a written protocol pertaining to the study and a verbal explanation about the purpose of the study. They were also informed that participation was voluntary, confidential and anonymous. Volunteers were also informed that they could withdraw from this study at any time without any repercussion to their treatments. Research participants were then asked to sign an informed consent form prior to their inclusion in the study.

### Instruments

#### HRQOLDA

The HRQoLDA Test is a quality-of-life assessment tool specific for drug abuse. It assesses the physical and psychosocial aspects of life through 20 five-choice items in a Likert-type scale, with choices being designated 1 to 5 points. The sum of the 20 scores represents quality of life, such that the higher the score, the better the quality of life.

DUQOL Scale: This scale is a specific measure of individual quality of life in drug users. It consists of 22 items relevant to the physical, social, psychological, occupational, and geographical reality of life, rated on a seven-point Likert-type scale ranging from 1 (very dissatisfied) to 7 (very satisfied). Higher test scores indicate better overall QOL.

WHOQOL-BREF Questionnaire. This generic questionnaire comprises 26 items, which measure the following broad domains: physical health, psychological health, social relationships, and environment. It consists of 24 questions chosen from the original WHOQOL-100 questionnaire, and 2 questions about satisfaction with Overall Quality of Life and General Health. The items have a five-point Likert-type scale ranging from 1 (lowest agreement) to 5 (highest agreement). Higher score indicates better QOL.

### Procedure

Five data collectors underwent a period of training and supervision by the principal investigator prior to administering questionnaires independently. The data collectors met regularly to address any queries and ensure each were following the same procedure.

The study participants completed the tests under minimal guidance from the trained examiners, who followed standardized instructional procedures. Interviews took place in a suitable room at the research sites mentioned above. Occasionally, specific questions not considered in the initial instruction procedure were answered on a one-to-one basis. Particular care was taken with non-native English speaking participants in order to ensure a satisfactory understanding.

### Analysis

The Rating Scale Model (RSM), a Rasch model for polytomous items, was used in the psychometric analysis [17]. The RSM is an extension to Rasch’s logistic model and is suitable for use when items are scored polytomously. In this study, the probability of response to an item is a logistic function determined by person HRQoL and the severity of item ‘δ’ at category ‘x’. This model transforms person and item raw scores to interval measures, which can be located on the same metric. Interpretations of the person’s HRQoL and item severity were carried out by transforming data to the “logit” scale.

When logit measures are compared between tests (tests of the same construct with different items), their probabilistic meaning is maintained but their substantive meanings may differ. Logit measures underlying different tests must be equated before the measures can be meaningfully compared [18].

Rasch analysis results are interpretable when data fit the model. The residual fit statistics used were the INFIT and OUTFIT indexes for each item and person. The INFIT index is sensitive to unexpected behavior affecting responses to items near the person's measure level (inliers) while the OUTFIT index is sensitive to unexpected behavior by persons on items far from the person's measure level (outliers) [18]. Both statistics can be reported by mean square residual (MnSq) and z-standardized mean square residual (Zstd). Acceptable values of MnSq statistics are between 0.5 and 1.5, where 1 is ideal [18]. Values larger than 1.5 indicate unmodeled noise or other source of variance in the data. Values less than 1.0 indicate overly predictable figures (overfit). In Zstd, 0 indicates that the model adequately predicts the observed data, and the range of −2 to +2 indicates an acceptable fit [18]. All analysis was conducted with WINSTEPS software version 3.64.2 [19].

**Fig. 1:**
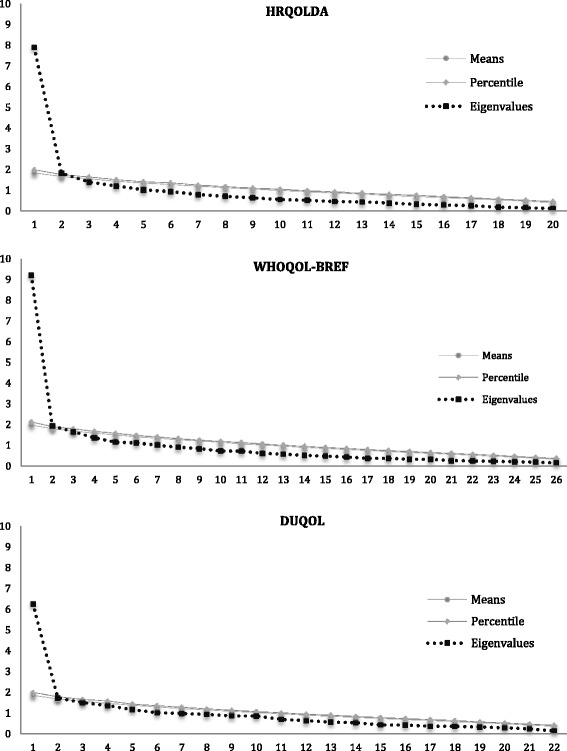
Parallel analysis graphics of HRQOLDA, WHOQOL-BREF and DUQOL

Considering that Rasch Models assumes unidimensionality, a parallel analysis was conducted to test dimensionality of HRQoLDA, the WHOQOL-BREF and the DUQOL. In parallel analysis, the mean eigenvalues and 95th percentile for the eigenvalues from random data were utilized as a baseline for determining dimensionality. All Factors with values greater than the baseline parameters (eigenvalues based on random data) were retained. The computer programs for parallel analysis used was developed by Patil, Singh, Mishra, & Donavan [20]. The results are compatible with the unidimensionality of the measures (see Fig. 1).

In order to check concordance between scores, a Reduction in Uncertainty (RiU) index was utilized [14]. This index reports information about statistical certainty of a dependent variable from a predictor variable. According to Dorans and Walker, RiU = 1-SQR (1-*r*^2^), where *r* is the correlation coefficient between both test scores [15]. When *r* = 0, there is a 0 % reduction; when *r* = 1, there is 100 % reduction. It is reasonable to expect at least 50 % of uncertainty reduction in one score resulted from the other score [15].

## Results

### Fit between Data and Model

Table 1 shows the fit statistics of the HRQOLDA, WHOQOL-BREF and DUQOL instruments. Only Item 19 for HRQOLDA test, Item 2 for DUQOL scale and Item 17 for WHOQOL-BREF questionnaire have infit (MnSq and Zstd) and outfit (MnSq and Zstd) values above the acceptable range. Fits statistics suggest acceptable goodness-of-fit to the RSM for three instruments.

**Table 1:** Item measures and fit statistics for the items

WHOQOL-BREF			INFIT		OUTFIT			DUQOL			INFIT		OUTFIT			HRQoLDA			INFIT		OUTFIT		
	ME	SE	MNSQ	ZEMP	MNSQ	ZEMP	R		ME	SE	MNSQ	ZEMP	MNSQ	ZEMP	R		ME	SE	MNSQ	ZEMP	MNSQ	ZEMP	R
1	.11	.09	.70	−1.0	.68	−1.1	.73	1	.03	.05	1.11	.4	1.16	.6	.49	1	.42	.08	1.10	.3	1.04	.1	.63
2	.31	.09	.69	−1.0	.75	−.8	.65	2	.30	.05	1.60	2.1	1.76	2.5	.27	2	.81	.09	1.05	.2	1.07	.2	.63
3	−.41	.09	1.68	1.6	1.75	1.8	.38	3	−.27	.06	1.00	.0	1.03	.1	.42	3	−.50	.09	.76	−.7	.70	−.7	.65
4	.31	.09	1.40	1.1	1.47	1.3	.35	4	.11	.05	1.12	.5	1.18	.7	.40	4	.90	.09	.82	−.5	.96	−.1	.64
5	−.06	.09	.69	−1.0	.66	−1.2	.70	5	−.06	.05	1.13	.5	1.11	.4	.51	5	.66	.08	.56	−1.4	.54	−1.4	.81
6	.07	.09	.89	−.3	.88	−.4	.63	6	.20	.05	.58	−2.1	.60	−1.9	.74	6	.41	.08	.68	−1.0	.62	−1.1	.76
7	.21	.09	.68	−1.1	.69	−1.0	.60	7	.07	.05	.90	−.4	.92	−.3	.54	7	.09	.08	.85	−.4	.80	−.5	.65
8	−.27	.09	.83	−.5	.87	−.4	.64	8	−.22	.05	1.30	1.0	1.39	1.2	.38	8	.48	.08	.76	−.7	.72	−.8	.72
9	−.18	.09	1.09	.3	1.08	.3	.49	9	.16	.05	.81	−.8	1.16	.6	.57	9	−1.08	.11	1.22	.4	1.30	.5	.43
10	.03	.09	.66	−1.1	.89	−.3	.64	10	−.28	.06	1.01	.1	1.03	.1	.47	10	.45	.08	.62	−1.2	.60	−1.2	.77
11	−.27	.09	1.11	.3	1.12	.4	.55	11	−.18	.05	1.32	1.1	1.23	.8	.49	11	−.84	.10	.89	−.2	.84	−.3	.55
12	.37	.09	1.31	.9	1.32	.9	.47	12	−.18	.05	.70	−1.2	.68	−1.2	.60	12	−.11	.08	1.69	1.6	1.51	1.1	.51
13	−.42	.09	.83	−.5	.82	−.5	.61	13	.16	.05	.88	−.5	.87	−.5	.58	13	−.13	.08	1.60	1.4	1.41	.9	.53
14	.14	.09	.94	−.2	.99	.0	.56	14	.36	.05	1.02	.1	1.01	.1	.50	14	−.33	.08	1.01	.0	1.09	.2	.62
15	-.51	.09	1.13	.4	1.11	.3	.57	15	-.09	.05	1.12	.5	1.09	.3	.46	15	.07	.08	.61	−1.2	.60	−1.2	.73
16	.45	.09	1.04	1	1.06	.2	.54	16	.12	.05	1.09	.4	1.12	.5	.43	16	−.06	.08	.56	−1.5	.53	−1.4	.78
17	.02	.09	.42	−2.2	.44	−2.2	.75	17	−.16	.05	.71	−1.2	.79	−.7	.45	17	−.19	.08	1.36	.9	1.53	1.1	.31
18	.36	.09	.75	−.8	.73	−.9	.68	18	.17	.05	1.18	.7	1.17	.7	.42	18	.10	.08	1.04	.1	1.12	.3	.56
19	.24	.09	.55	−1.6	.55	−1.6	.77	19	−.12	.05	1.06	.2	1.05	.2	.45	19	.06	.08	1.94	2.1	2.09	2.2	.32
20	.24	.09	1.04	.1	1.03	.1	.62	20	−.06	.05	1.14	.5	1.15	.6	.42	20	−1.21	.12	1.46	.7	2.25	1.4	.13
21	.31	.09	1.47	1.3	1.54	1.4	.40	21	.02	.05	.70	−1.3	.68	−1.4	.63								
22	−.01	.09	1.23	.7	1.24	.7	.46	22	−.06	.05	.59	−1.9	.57	−1.9	.67								
23	−.38	.09	1.37	1.0	1.34	.9	.54																
24	−.69	.10	1.24	.6	1.24	.6	.37																
25	−.28	.09	1.67	1.7	1.63	1.6	.43																
26	.30	.09	.77	−.8	.75	−.8	.68																
ITEM summary								ITEM summary								ITEM summary							
MEAN	.00	.09	1.01	−.1	1.02	.0		MEAN	.00	.05	1.00	−.1	1.04	.1		MEAN	.00	.09	1.03	−.1	1.07	.0	
S.D.	.31	.00	.33	1.0	.34	1.0		S.D.	.18	.00	.25	1.0	.26	1.0		S.D.	.57	.01	.39	1.0	.48	1.0	
PERSONS summary								PERSONS summary								PERSONS summary							
MEAN	.02	.20	1.02	−.1	1.02	−.1		MEAN	.14	.13	1.02	−.1	1.04	.0		MEAN	.29	.22	1.03	.0	1.07	1	
S.D.	.72	.04	.48	1.0	.48	1.0		S.D.	.37	.04	.42	1.0	.43	1.0		S.D.	.73	.06	.49	1.0	.59	1.0	

ME: Items Measures; SE: Standard Errors; INFIT: information-weighted fit statistic; OUTFIT: is an outlier-sensitive fit statistic; MNSQ: Mean Square Residual; ZEMP: z-Standardized Mean

### Concordance among the DUQOL, HRQOLDA and WHOQOL-BREF Scores

**Fig. 2:**
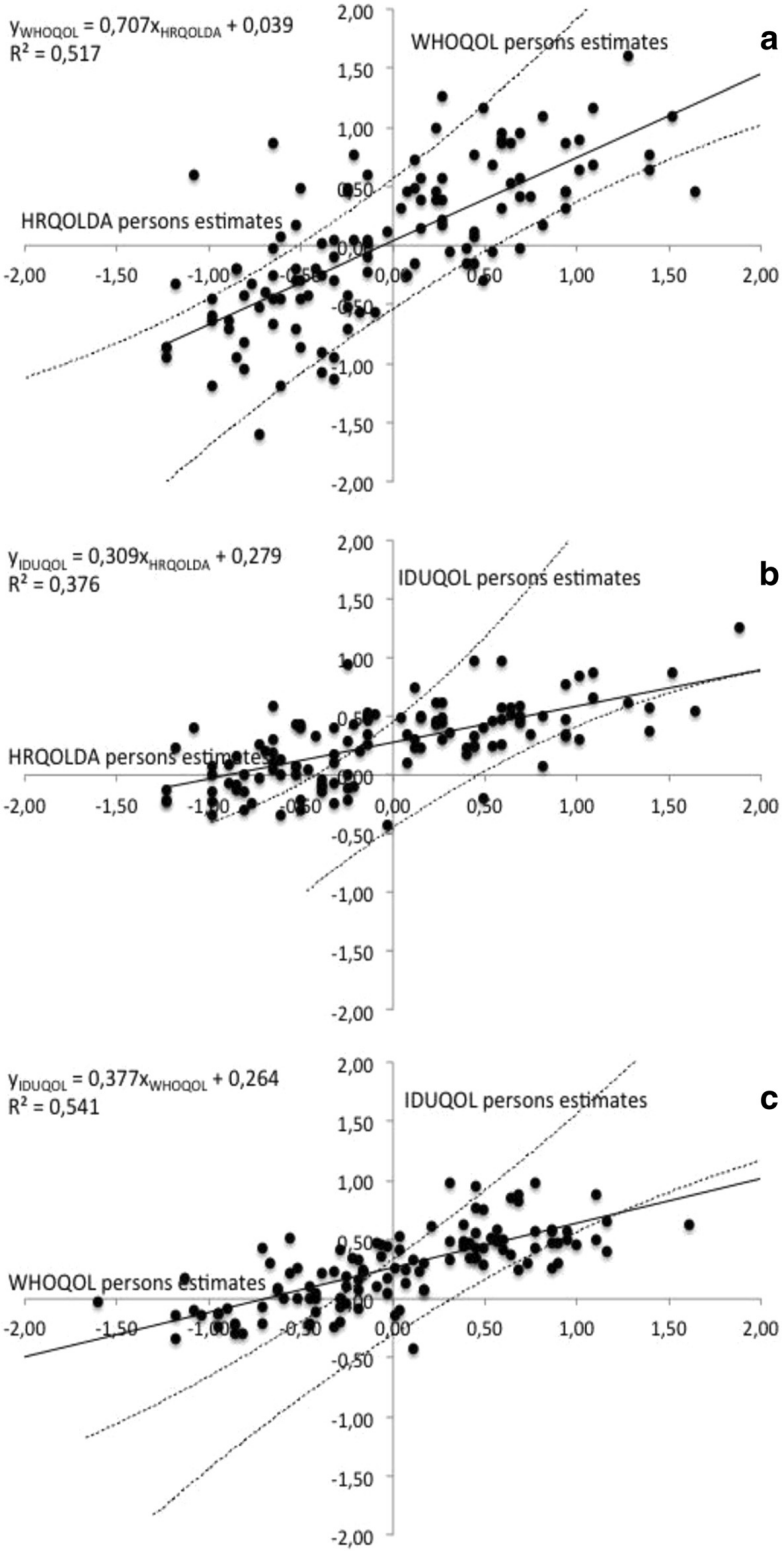
Common person equating: HRQOLDA test and WHOQOL-BREF Questionnaire (**a**), HRQOLDA test and DUQOL scale (**b**), and WHOQOL-BREF Questionnaire and DUQOL scale (**c**). Dots represent persons. The black line represents the empirical regression line. The dotted lines represent the estimates’ 95 % CI

Common-person’s equating of two instruments involves an assessment of the invariance of person’s estimates of a single-sample. The RSM person’s estimates (logit scale) for each person in both tests are displayed in a scatterplot; the plots should fall on a single line with allowance made for the modeled standard error pairs for each person’s estimates [21]. Figure 2 shows the scatterplot of RSM person’s estimates of the HRQoLDA and the WHOQOL-BREF against each other, the scatterplot of RSM person’s estimates of the HRQoLDA and the DUQOL in similar comparison, and the scatterplot of RSM person’s estimates of the WHOQOL-BREF and the DUQOL against each other as well. The 95 % confidence band provides a means to evaluate the extent to which the two tests are measuring the same construct within a reasonable degree of measurement error.

Before displaying the scatterplot, following the recommended steps for common subject equating [21], the mean of the ability estimates for each person in each instrument was achieved. Subsequently, the differences between the means were computed - HRQoLDA was compared by using as the point of reference 0.00 logits: the HRQOLDA mean (0.29 logits), and WHOQOL-BREF mean (0.20 logits) were contrasted with the HRQoLDA mean and DUQOL mean (0.14 logits). The person ability estimates by the mean difference (add the difference to each estimate) were recalculated: HRQoLDA mean (0.00 logits), WHOQOL-BREF mean (0.04 logits), and DUQOL mean (0.28 logits).

In Fig. 2, about 80 % of all plots are located inside the confidence interval, whereas in Figs. 2, about 60 % of all plots are located inside the confidence interval. The degree of concordance of health-related quality of life measures among the instruments varies. Under ideal circumstances of perfect concordance, the empirical regression line should have a slope of 1 and an intercept of 0. The slope of the empirical regression line is 0.707 and the intercept 0.039, when HRQoLDA and WHOQOL-BREF are compared (R^2^ = 0.517). The slope of the empirical regression line is 0.309 and the intercept 0.279, when HRQoLDA and DUQOL are compared (R^2^ = 0.376). Correlations between HRQoLDA and WHOQOL-BREF and between HRQoLDA and DUQOL scores were 0.719 and 0.613, respectively. The RiU index between HRQoLDA and WHOQOL-BREF was 30.4 % and between HRQoLDA and DUQOL was 20.9 %.

### Severity item estimates

The analyses of the items yielded a severity item range (Table 1) for HRQoLDA test of −1.21 to 0.90 logits (x̄ = 0; SD = 0.57); for WHOQOL-BREF questionnaire the respective values were −0.69 to 0.46 logits (x̄ = 0; SD = 0.31), and for DUQOL scale the values were - 0.28 to 0.36 logits (x̄ = 0; SD = 0.18).

**Fig. 3:**
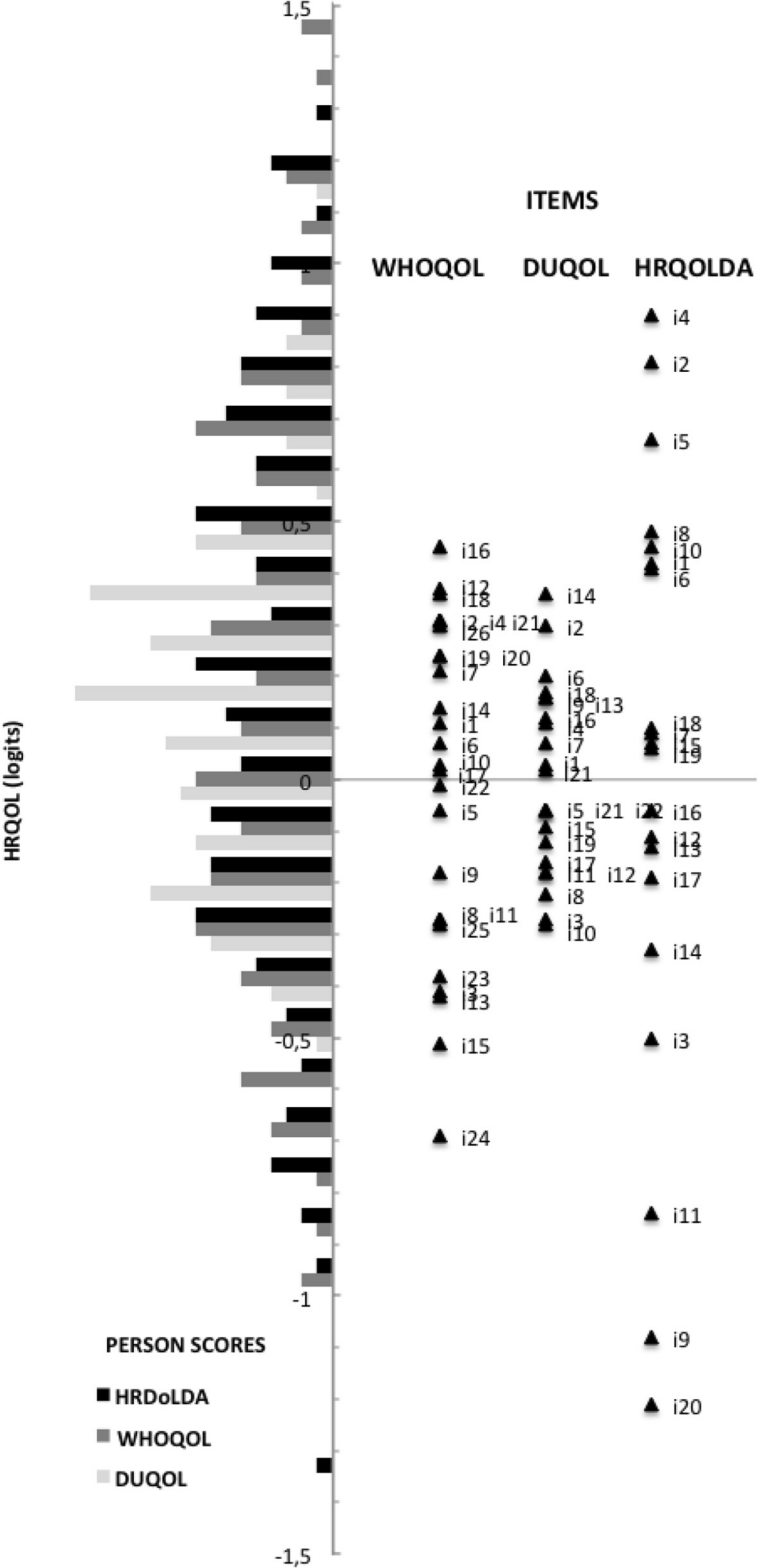
The HRQOLDA, WHOQOL-BREF and DUQOL item maps

The map of persons and items (Fig. 3) shows the combined position of the HRQoLDA, the DUQOL and the WHOQOL-BREF severity of items on the HRQoL continuum (right side). This map shows the item hierarchy that measured by the instruments. The HRQoL continuum is shown on the left of the plot, while on the right of plot the items appear in an order according to their severity values on the HRQoL continuum. It can be seen in Fig. 3 that the range of severity of items is greater in the HRQoLDA in comparison with the WHOQOL-BREF and the DUQOL. The DUQOL shows the lowest range of severity of items. The measurements of the persons by test are displayed on the left side are. The map of persons and items is useful for comparing the range and position of the items and can be also utilized to measure person’s distributions. In order to develop a proper measure for all persons, the items must cover all the areas on the HRQoL continuum.

### Reliability, separation and standard errors of measurement

In general, with Rasch models, an analysis is carried out to compute a separation index for and for items, instead of reliability coefficients. Person Separation Index (PSI) is used to classify people; it represents the number of statistically different performance strata that the instrument can detect in the sample. Low person’s separation (less than 2) indicates that the instrument could be not sensitive enough to classify between people with high and low ability [18]. The HRQoLDA, the WHOQOL-BREF and the DUQOL person separation index were of 3.02, 3.09 and 2.58, respectively.

Item Separation Index (ISI) is used to demonstrate item hierarchy. The hierarchy of item severities is particularly important given that the planned items severity in the test content specifications can be compared with the order estimated from the data [18]. Low item separation (less than 3) implies that the person’s sample is not large enough to confirm the items hierarchy of the instrument (and that the test has no item with high, medium or low severity) [18]. The values of the HRQoLDA, WHOQOL-BREF and DUQOL item separation indexes were respectively 6.41, 3.30 and 3.28.

**Fig. 4:**
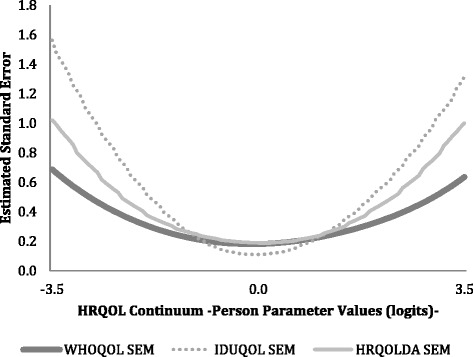
Estimated errors standard of measurement of HRQOLDA, WHOQOL-BREF and DUQOL instruments along the HRQOL continuum

The Rasch reliability (of persons) is comparable to the traditional reliability of the test. The HRQoLDA, WHOQOL-BREF and DUQOL reliability coefficients were 0.90, 0.92 and 0.87, respectively. In Rasch models, each ability estimate has an associated standard error of measurement (SEM). The estimates of the SEM are displayed in Fig. 4. The general shape of the three curves shows that the SEM is not constant across the range of tests. The standard error of the HRQoLDA, WHOQOL-BREF and DUQOL measures are smaller in the center of the HRQoL continuum and bigger on the extremes. The mean of SEM in the interval [−2.5, −1.6] was 0.42 logits for the HRQoLDA, 0.34 logits for the WHOQOL-BREF and 0.60 logits for the DUQOL. The mean of SEM in the interval [−0.5, 0.4] was 0.19 logits for the HRQoLDA, 0.18 logits for the WHOQOL-BREF and 0.12 logits for the DUQOL. The mean of SEM in the interval [1.5, 2.4] was 0.42, 0.31 and 0.52 logits for the HRQoLDA, the WHOQOL-BREF and the DUQOL respectively.

## Discussion

The HRQoL is considered one of the essential measures required for the optimal assessment of the results of treatment interventions in drug dependence. Considering that the evaluation of quality of life of substance users is usually performed with different tests, the objective of this study was to establish the concordance among the HRQoLDA, the DUQOL (which is a specific assessment tool for substance use), and the WHOQOL-BREF (a widely used generic quality of life measurement instrument). Although there are comparative studies with different QoL assessment tools in the context of several specific disorders [22, 23], to the authors’ knowledge, this is the first time that results of a comparative study with specific HRQoL assessment tools for substance use is presented. In addition, in this study an IRT approach was followed to establish the concordance of generic and specific QoL assessment tools.

The results here presented indicate moderate to high correlation among all three tests. The results also demonstrated a higher concordance between the HRQoLDA and WHOQOL-BREF than between the HRQoLDA and the DUQOL. However, it cannot be established unequivocally, on the basis of the above-presented results, that the scores of these tools are concordant, given the low values of RiU indexes. It has been advocated that, if a variable cannot reduce uncertainty by at least 50 %, it is unlikely that the predictor can serve as a valid surrogate, via concordance, for the score being predicted [15].

From a psychometric perspective, the advantages of IRT models and Rasch models, in particular, are obtained when data fit to model. The results have shown that data from all three tests fit adequately the RSM. Therefore, in all three tests, the pattern of responses produced by participants follow a common conceptual logic. One of the advantages of the RSM is that, whenever data fit the model, measurement levels area transformed from an ordinal to an interval scale. The construct of HRQoL is usually explored as an outcome in efficacy and effectiveness analyses. Thus, working with scores in an interval scale, instead of an ordinal one, is preferable both for the purpose of improving the interpretation of scores produced by research participants, as in this particular case, and for conducting the statistical analysis of data.

On the map of the scale’s items, the location to each item can be identified on the HRQoL continuum. The HRQoLDA is a test that evaluates the widest range within the HRQoL continuum, followed by the WHOQOL-BREF and by the DUQOL. This distribution of items is important to avoid floor and ceiling effects observed in previous studies [9]. It has been noticed that the HRQoLDA is the most effective test whenever a low or high severity of impairment is predominant in a given sample. Nevertheless, the HRQoLDA can also perform with sensitivity in pre-post study designs on any occasion a significant impact of treatment is expected. Notwithstanding, it has also been shown that the WHOQOL-BREF is sensitive to detect measurement changes in pre-post study designs [24].

In terms of accuracy, the DUQOL is the test that has demonstrated a lower error of measurement (higher accuracy in the central area of the continuum). Contrarily, the WHOQOL-BREF revealed more accurate scores on the extreme areas of the continuum, which was slightly superior and more accurate than the HRQoLDA on the same area of the continuum. In terms of reliability, all three tests reveal similar and adequate results. The Person Separation Index (PSI) was similar in both the WHOQOL-BREF and the HRQoLDA tests, while superior than the PSI observed for the DUQOL. Yet, according to the proposed psychometric parameters in this field, all three tests perform adequately for differentiating between individuals whose scores are located at different points along the continuum of the HRQoL construct. The three tests showed adequate values of Item Separation Index (ISI). The ISI was significantly higher in the HRQoLDA, which indicates that the HRQoLDA items perform at a superior level along the HRQoL continuum.

In summary, the DUQoL is the most accurate instrument for the central area of the distribution of scores, but it is also the test with the narrowest range of item measures. Such characteristic can limit its usefulness in heterogeneous patient samples (especially if individuals present with extreme scores). The WHOQOL is the best instrument for comparing the scores among groups of patients and for general population use, given its conception and design as a generic instrument. Finally, the results of this study show that the HRQoLDA may be the most suitable tool to be utilized in efficiency and effectiveness studies. The HRQoLDA is the instrument with a widest range of item measures; it presents with less probability of floor and ceiling effects. This is particularly useful in the detection of clinically significant changes for more extreme scores.

One of the limitations of this study relates to the sample size (121 participants). In spite of this limitation, it has been demonstrated in simulation studies that Rasch analyses are able to produce precise estimations (items and persons parameters) in analysis with samples with 100 or more participants [25].

## Conclusions

The results of this study reveal a high correlation between the scores of the HRQOLDA and WHOQOL-BREF questionnaire and a moderate correlation between the scores of the HRQOLDA and DUQOL questionnaire. There was a higher concordance (like a procedure of transformations between the scores from one test to another) between the HRQoLDA and WHOQOL-BREF than between the HRQoLDA and the DUQOL. However, it cannot be established unequivocally that the scores of these tools are concordant. In this study, the utility of the application of the Rasch model was demonstrated in the process of producing an empirical benchmark for the selection of measurement tools to be used in the context of health care and research.
